# Spermatozoon ultrastructure in two monorchiid digeneans

**DOI:** 10.7717/peerj.2488

**Published:** 2016-09-20

**Authors:** Yann Quilichini, Abdoulaye J.S. Bakhoum, Jean-Lou Justine, Rodney A. Bray, Cheikh T. Bâ, Bernard Marchand

**Affiliations:** 1 GEM - Service d’Etude et de Recherche en Microscopie Electronique (SERME), UMR 6134 –SPE, CNRS –University of Corsica, Corte, Corsica, France; 2 Laboratory of Evolutionary Biology, Ecology and Management of Ecosystems, University Cheikh Anta Diop of Dakar, Dakar, Senegal; 3 ISYEB, Institut de Systématique, Évolution, Biodiversité (UMR7205 CNRS, EPHE, MNHN, UPMC), Muséum National d’Histoire Naturelle de Paris, Sorbonne Universités, Paris, France; 4 Department of Life Sciences, National History Museum of London, London, United Kingdom

**Keywords:** Sperm, Ultrastructure, Digenea, Monorchiidae, *Opisthomonorchis*, *Paramonorcheides*, TEM

## Abstract

Spermatological characteristics of species from two monorchiid genera, *Opisthomonorchis* and *Paramonorcheides*, have been investigated, for the first time, by means of transmission electron microscopy. The ultrastructural study reveals that the mature spermatozoon of *Opisthomonorchis dinema* and *Paramonorcheides selaris* share several characters such as the presence of two axonemes of different lengths showing the 9+“1” pattern of the Trepaxonemata, a nucleus, two mitochondria, two bundles of parallel cortical microtubules, external ornamentation of the plasma membrane, spine-like bodies, granules of glycogen and similar morphologies of the anterior and posterior extremities. The slight differences between the male gamete of *O. dinema* and *P. selaris* are the length of the first axoneme and the position of the second mitochondrion. This study also elucidates the general morphology of the spermatozoon in all monorchiid species described so far, which corresponds to a unique spermatozoon type. Other interesting finds concern the spermatological similarities between monorchiid spermatozoa and the mature spermatozoon reported in the apocreadiid *Neoapocreadium chabaudi*. These similarities allow us to suggest a close phylogenetical relationship between the Monorchiidae and the Apocreadiidae, although more studies are needed, especially in the unexplored taxa.

## Introduction

The description of ultrastructural characteristics of the mature spermatozoon of the Platyhelminthes, and in particular the Digenea, has recently proved to be an interesting tool for phylogenetic purposes (Justine, 1995; Levron et al., 2010; Miquel et al., 2013; Bakhoum et al., 2012a; Bakhoum et al., 2012b; Bakhoum et al., 2015a; Bakhoum et al., 2015b; Ndiaye et al., 2015a; Ndiaye et al., 2015b). The Monorchioidea comprise two families of trematodes namely, the Lissorchiidae and the Monorchiidae (Bray, 2008). Among the latter, which includes 40 valid genera (Madhavi, 2008), only two species, *Monorchis parvus* and *Helicometroides atlanticus* (Levron, Ternengo & Marchand, 2004a; Diagne et al., 2015) have been explored for spermatological characteristics. Moreover, the mature spermatozoon of *M. parvus* and *H. atlanticus* exhibit some differences especially in their respective anterior extremity.

The aim of this work was to bring more spermatological descriptions from two additional genera, *Opisthomonorchis* and *Paramonorcheides*, in order to elucidate the general morphology of the spermatozoon in the Monorchiidae. In addition, the mature spermatozoon of the monorchiids was compared to those of the other digeneans with a brief comment on its phylogenetic relevance.

## Materials and Methods

Fish were bought dead, but very fresh, at the fishmarket in Nouméa, New Caledonia. Specimens of *Opisthomonorchis dinema* Bray & Justine, 2013 were collected from the digestive tract of *Carangoides dinema* Bleeker (Perciformes, Carangidae) caught off Nouméa, New Caledonia, from the same fish specimen (MNHN JNC3224) as the paratypes of the species (Bray & Justine, 2013). Specimens of *Paramonorcheides selaris* Lakshmi & Madhavi, 2009 were collected from the digestive tract of *Selar crumenophthalmus* (Bloch, 1793) (Perciformes, Carangidae) caught off Nouméa, New Caledonia, on 10 Sept. 2009 (fish specimen MNHN JNC 3043).

Digenean specimens were fixed in cold (4°C) 2.5% glutaraldehyde in 0.1 M sodium cacodylate buffer at pH 7.2, rinsed in 0.1 M sodium cacodylate buffer at pH 7.2, post-fixed in cold (4°C) 1% osmium tetroxide in the same buffer for 1 h, dehydrated in ethanol and propylene oxide series, embedded in Spurr resin and polymerized at 60°C for 24 h. Ultrathin sections (60–90 nm) of the seminal vesicle were obtained on an ultramicrotome (Power tome PC, RMC Boeckeler^®^). The sections were placed on 300 and 200 mesh copper grids and stained with uranyl acetate and lead citrate according to Reynolds (1963) methodology. To locate glycogen granules, the Thiéry technique (1967) was also used in several sections placed on gold grids.

All grids were examined on a Hitachi H-7650 transmission electron microscope, operating at an accelerating voltage of 80 kV, in the “Service d’Étude et de Recherche en Microscopie Électronique” of the University of Corsica (Corte, France).

## Results

**Figure 1:**
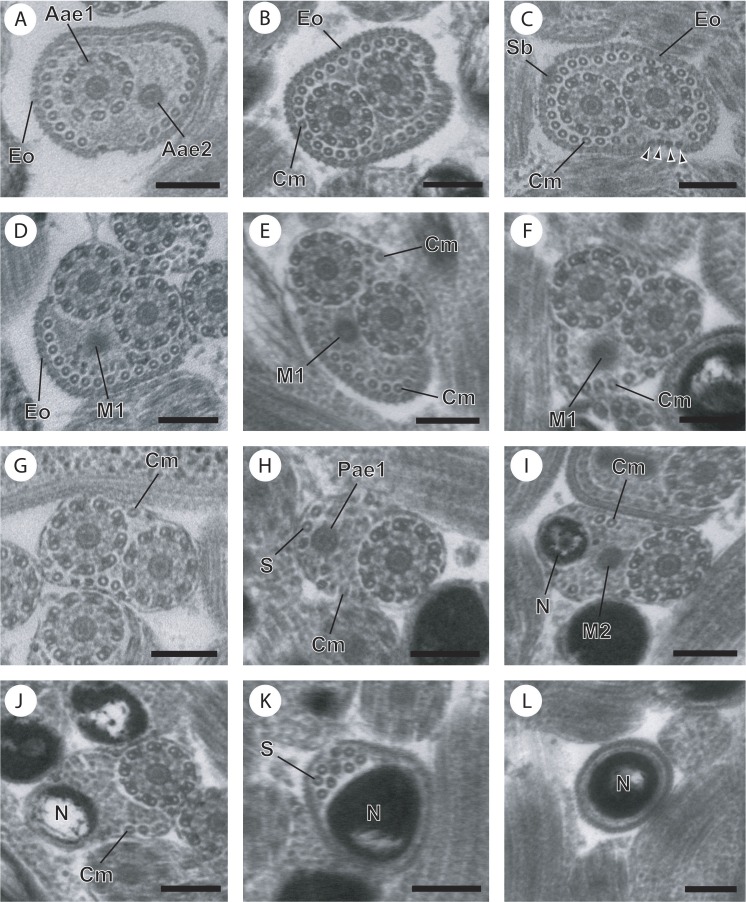
Cross-sections of the mature spermatozoon of *Opisthomonorchis dinema* observed in TEM. (A) Region I showing the anterior extremities of the two axonemes, the external ornamentation and cortical microtubules; (B and C) middle part of the region I exhibiting (B) both formed axonemes, the external ornamentation associated with cortical microtubules, spine-like body and attachment zones (C); (D and E) posterior part of the region I characterized by the appearance of the first mitochondrion and the cortical microtubule; (F and G) region II or transitional areas showing (F) the posterior part of the first mitochondrion, the axonemes and a decreasing number of cortical microtubules from about 10 to 5; (H) anterior part of the region III characterized by the disorganization of the first axoneme; (I) middle part of the region III characterized by the appearance of the second mitochondrion and the nucleus; (J) posterior part of the region III characterized by only the nucleus, one axoneme and cortical microtubules; (K and L) region IV corresponding to the posterior spermatozoon extremity. Note the disorganization of the second axoneme (K). Scale bars: 0.2 µm. Arrowheads indicate attachment zones. Aae1, anterior extremity of first axoneme; Aae2, anterior extremity of second axoneme; Cm, cortical microtubules; Eo, external ornamentation of the plasma membrane; M1, first mitochondrion; M2, second mitochondrion; N, nucleus; Pae1, posterior extremity of axoneme 1; S, singlet of microtubule; Sb, spine-like body.

**Figure 2:**
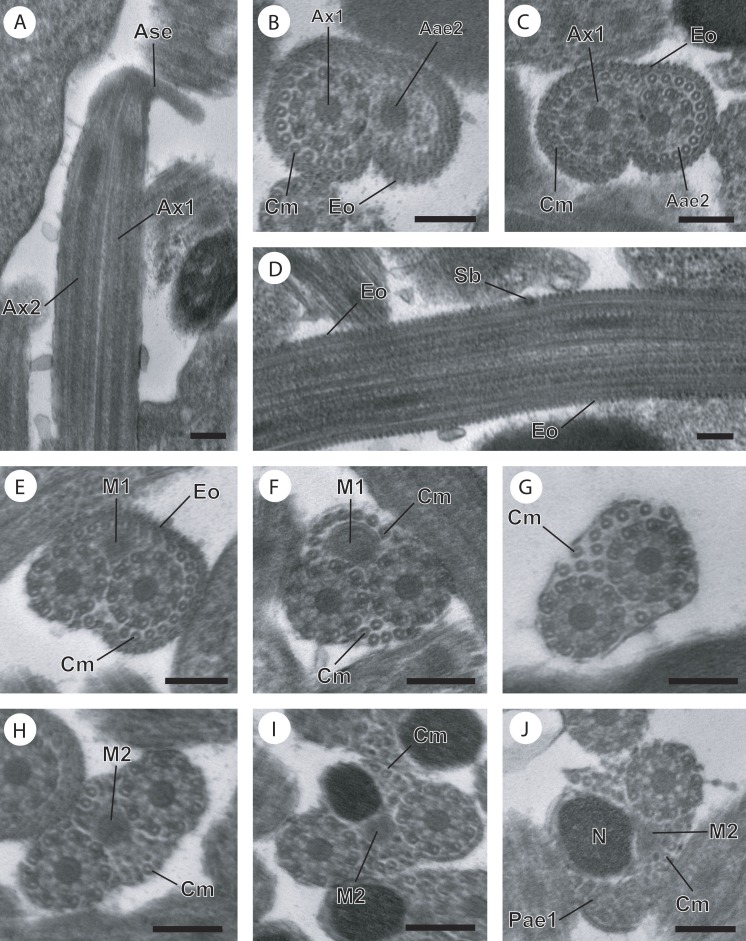
Regions I−III of mature spermatozoon of *Paramonorcheides selaris* observed in TEM. (A–C) Longitudinal and cross-sections of region I corresponding to the anterior spermatozoon extremity; (D) longitudinal section in the ornamented area showing the spine-like body interrupting the external ornamentation of the plasma membrane; (E) cross-section in the posterior part of the region I characterized by the appearance of the first mitochondrion; (F and G) consecutive cross-sections in region II or transitional area showing only the axonemes, the first mitochondrion and a decrease of the cortical microtubules number; (H) anterior part of region III exhibiting the second mitochondrion; (I and J) cross-sections of middle part of region III showing the appearance of the nucleus. Note the disorganisation of the first axoneme (J). Scale bars: 0.2 µm. Aae2, anterior extremity of second axoneme; Ase, anterior spermatozoon extremity; Ax1, first axoneme; Ax2, second axoneme; Cm, cortical microtubules; Eo, external ornamentation of the plasma membrane; M1, first mitochondrion; M2, second mitochondrion; N, nucleus; Pae1, posterior extremity of the first axoneme; Sb, spine-like body.

**Figure 3:**
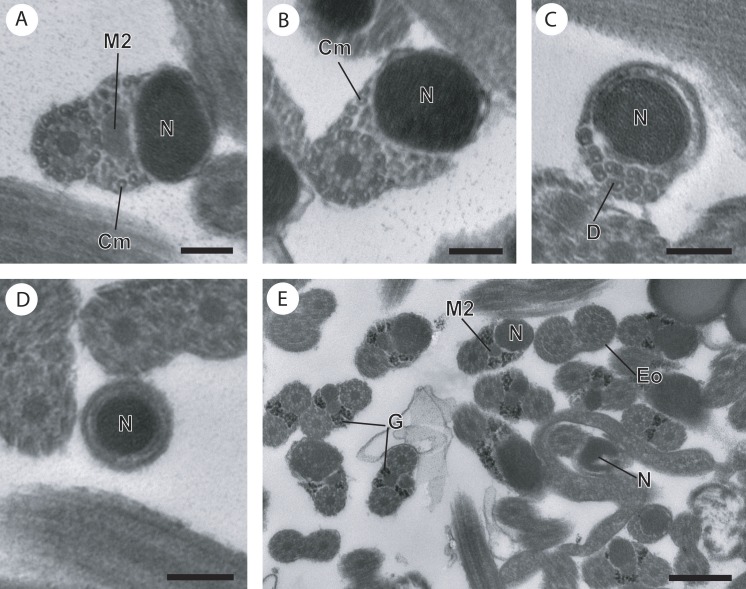
Cross-section of regions III and IV of the mature spermatozoon of *Paramonorcheides selaris* observed in TEM. (A) Posterior part of the region III characterized by the nucleus, the second axoneme, the second mitochondrion and cortical microtubules; (B and C) region IV characterized by the nucleus, the second axoneme which disorganizes progressively, and the posterior extremity of the last cortical microtubules; (D) posterior part of region IV containing only the nucleus; (E) positive test of Thiéry for labelling of glycogen. Scale bars: 0.2 µm (A–D), 0.5 µm (E). Cm, cortical microtubules; D, doublets of microtubule; Eo, external ornamentation of the plasma membrane; G, granules of glycogen; M2, second mitochondrion; N, nucleus.

**Figure 4:**
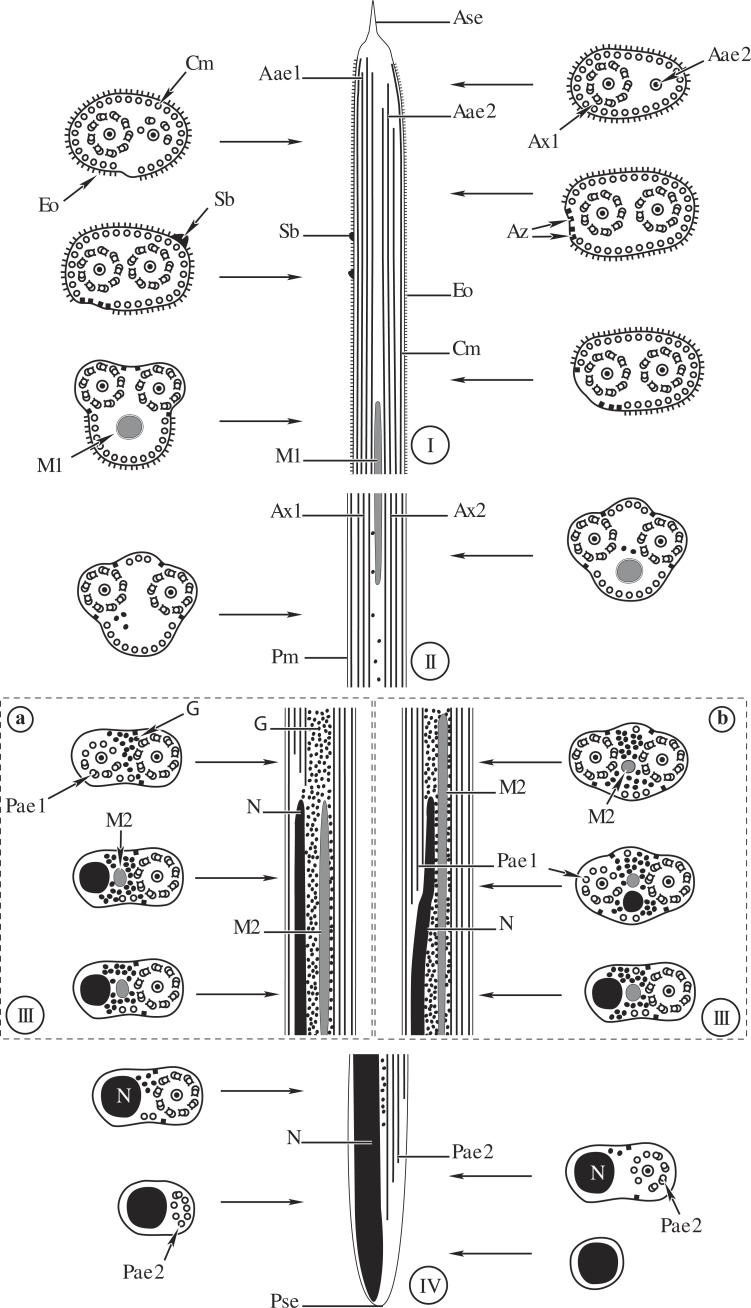
Comparative schematic reconstruction of the mature spermatozoa of *Opisthomonorchis dinema* (A) and *Paramonorcheides selaris* (B). Aae1, anterior extremity of first axoneme; Aae2, anterior extremity of second axoneme; Ase, anterior spermatozoon extremity; Ax1, first axoneme; Ax2, second axoneme; Az, attachment zone; Cm, cortical microtubules; Eo, external ornamentation of the plasma membrane; G, granules of glycogen; M1, first mitochondrion; M2, second mitochondrion; N, nucleus; Pae1, posterior extremity of the first axoneme; Pae2, posterior extremity of the second axoneme; Pm, plasma membrane; Pse, posterior spermatozoon extremity; Sb, spine-like body.

The organization of the mature spermatozoon of *Opisthomonorchis dinema* and *Paramonorcheides selaris* is described after observations of cross- and longitudinal sections (Figs. 1–4). Thus, in both monorchiid species four regions, with distinctive ultrastructural characteristics, are evident, from the anterior to the posterior spermatozoon extremities. The granules of glycogen present in *O. dinema* and *P. selaris* spem cells were detected by the cytochemical test of Thiéry (Fig. 3E for *P. salaris*).

Region I, corresponding to the anterior extremity of the mature spermatozoon, is characterized by the presence of the first axoneme accompanied by the anterior extremity of the second axoneme, cortical microtubules and external ornamentation of the plasma membrane. In *O. dinema* the anterior spermatozoon tip exhibits both anterior extremities of the axonemes, surrounded by a layer of cortical microtubules (about 26) and external ornamentation (Figs. 1A, 1B and 4I). In *P. selaris* longitudinal and cross-sections of the anterior spermatozoon tip show that it tapers to a point (Fig. 2A), and that it contains the anterior extremities of both axonemes and the cortical microtubules (about 26) (Fig. 2C) as well as exhibiting external ornamentation of the plasma membrane (Figs. 2B, 2C and Fig. 4I). In the middle part of the region I, when both axonemes are formed, spine-like bodies appear interrupting the external ornamentation of the spermatozoa of *O. dinema* and *P. selaris* (Figs. 1C, 2D and 4I). The posterior part of the region I is characterized, for each species, by the presence of external ornamentation of the plasma membrane associated with cortical microtubules and the first mitochondrion (Figs. 1D, 1E, 2E and 4I). No glycogen granule have been highlighted by the Thiéry’s method in this region.

Region II corresponds to the transitional area before the nuclear region. For both *O. dinema* and *P. selaris* spermatozoa, it is characterized by the absence of external ornamentation and the presence of the axonemes and the first mitochondrion (Figs. 1F, 2F and Fig. 4II). In addition, for each monorchiid species, the posterior part of the region II contains a reduced number of cortical microtubules: about 5 in *O. dinema* (Fig. 1G) and 13 in *P. selaris* (Fig. 2G). Only few granules of glycogen are observed in this region.

Region III exhibits several ultrastructural characteristics that distinguish the mature spermatozoa of *O. dinema* (Figs. 3A and 4IIIA) from those of *P. selaris* (Figs. 3B, 4IIIB):

– In the anterior part of the region III, the spermatozoon is characterized by the disorganization of the first axoneme in *O. dinema* (Figs. 1H, 4IIIA), whereas in *P. selaris* the two axonemes and the second mitochondrion are observed (Figs. 2H, 4IIIB).
– The middle part of the region III is characterized in *O. dinema* by the presence of only one axoneme, the nucleus, few cortical microtubules and the second mitochondrion (Figs. 1I and 4IIIA). In contrast, in *P. selaris*, the second mitochondrion, the nucleus and few cortical microtubules were accompanied by the two axonemes (Figs. 2I, 4IIIB). Note that this part corresponds to the disorganization of the first axoneme in *P. selaris* (Figs. 2J and 4IIIB).
– In the posterior part of the region III, the second mitochondrion, accompanied by one axoneme, the nucleus and cortical microtubules is observed in both *O. dinema* (Figs. 1I and 4III) and in *P. selaris* (Fig. 3A, 4III).

This region is characterized, for the two species, by a high number of glycogen granules.

Region IV represents the posterior spermatozoon extremity. When the second mitochondrion disappears in both *O. dinema* and *P. selaris* mature spermatozoa, cross-sections exhibit only one axoneme, the nucleus and very few cortical microtubules (Figs. 1J, 3B and 4IV). Moreover, for each species, the second axoneme disorganizes exhibiting doublets and singlets of microtubules (Figs. 1K, 3C). Consequently, the posterior spermatozoon tips in *O. dinema* and *P. selaris* show only the nucleus (Figs. 1L, 3D and 4IV).

For the two species, only few glycogen granules have been observed in the anterior part of this region.

## Discussion

The mature spermatozoon described in *Opisthomonorchis dinema* and *Paramonorcheides selaris* possesses two axonemes of the 9+“1” trepaxonematan pattern, two mitochondria, a nucleus, two bundles of parallel cortical microtubules, an external ornamentation of the plasma membrane, spine-like bodies and granules of glycogen. In addition to these ultrastructural characters, the male gamete of *O. dinema* and *P. selaris* exhibits similar anterior and posterior spermatozoon morphologies.

Within the Monorchiidae most of these spermatological characters were described previously in *Monarchus parvus* and *Helicometroides atlanticus* (Levron, Ternengo & Marchand, 2004a; Diagne et al., 2015). Thus, the characters of the mature spermatozoon, its anterior and posterior morphologies were used here in comparative studies between the different species (Table 1).

**Table 1:** Comparative ultrastructural characteristics of the spermatozoon in the Monorchiidae. Ase, anterior spermatozoon extremity; Ax, axoneme; Cm, cortical microtubules; Edm, electron-dense material; Eo, external ornamentation of the plasma membrane; M, number of mitochondria; MCm, maximum number of cortical microtubules; N, nucleus; TAx, type of axoneme; TEo, type of external ornamentation according to Quilichini et al., (2011a); Psc, posterior spermatozoon character; Sb, spine-like bodies; +/−, 2013, presence/absence of considered character.

Parasites species	Spermatological characteristics								References
	TAx	Ase	Eo	TEo	Sb	MCm	M	Psc	
*Helicometroides atlanticus*	9+“1”	2Ax+Edm	+	2	−	35	2	N	Diagne et al. (2015)
*Monorchis parvus*	9+“1”	2Ax+Eo	+	1	−	25	2	N	Levron, Ternengo & Marchand (2004a)
*Opisthomonorchis dinema*	9+“1”	2Ax+Eo	+	1	+	28	2	N	Present study
*Paramonorcheides selaris*	9+“1”	2Ax+Eo	+	1	+	26	2	N	Present study

### General organisation of the mature spermatozoon in the Monorchiidae

From the anterior part to the posterior part of the gametes, six characteristics are presented here to describe the architecture of the mature spermatozoa in Monorchiidae. Moreover, monorchiid spermatozoa are compared to those reported from the other digenean species.

#### Type of axoneme

The structure of the axoneme with 9+“1” pattern of Trepaxonemata (Ehlers, 1984) has been observed in *O. dinema* and *P. selaris* as described previously in *M. parvus* and *H. atlanticus* (Table 1). A similar morphology of the axoneme has been reported in most digenean species (Quilichini et al., 2011a; Foata et al., 2012; Miquel et al., 2013; Ndiaye et al., 2012; Ndiaye et al., 2015a) with the exception of schistosomes in which the 9+“1” special pattern was described and some didymozoid species showing the 9+0 pattern (Justine & Mattei, 1983; Justine, Jamieson & Southgate, 1993; Yang, Dong & Jiang, 2003).

Their different length is another aspect related to axonemes. In both *O. dinema* and *P. selaris*, the posterior extremity of the axoneme 2 is longer than that of the first one. Moreover, the spermatozoon of *O. dinema* is distinguished by the first axoneme that does not reach the nuclear region, while in *P. selaris* the first axoneme is observed in the nuclear area like in *M. parvus* and *H. atlanticus* (Levron, Ternengo & Marchand, 2004a; Diagne et al., 2015).

#### Anterior spermatozoon morphology

The anterior spermatozoon extremity of *O. dinema* and *P. selaris* contains two axonemes, cortical microtubules and external ornamentation of the plasma membrane. Within the Monorchiidae, an anterior spermatozoon extremity showing two axonemes, cortical microtubules and external ornamentation has been reported in *Monorchis parvus* (Levron, Ternengo & Marchand, 2004a). Whereas in the other monorchiid species studied so far, namely *H. atlanticus* (Diagne et al., 2015), only electron-dense material and two axonemes have been observed in the anterior spermatozoon extremity.

Mature spermatozoa showing two axonemes and external ornamentation in their anterior extremities have also been reported in spermatozoa belonging to some digenean species such as the apocreadiid *Neoapocreadium chabaudi* (Kacem et al., 2010), the pronocephalids *Pleurogonius truncatus* and *Cricocephalus albus* (Ndiaye et al., 2011; Ndiaye et al., 2012) and the mesometrids *Centroderma spinosissima* and *Wardula capitellata* (Bakhoum et al., 2012b; Bakhoum et al., 2013). In the two latter families, the presence of a lateral expansion is described in the anterior spermatozoon extremity of the studied species.

Other types of spermatozoa showing two axonemes in their anterior extremities have been reported in several digenean species such as the deropristid *Deropristis inflata* (Foata, Quilichini & Marchand, 2007), the omphalometrid *Rubenstrema exasperatum* (Bakhoum et al., 2011) or the pleurogenids *Pleurogenes claviger*, *Pleurogenoides medians* and *Prosotocus confusus* (Miquel et al., 2013).

#### External ornamentation and its location

In all monorchiid species studied, the presence of external ornamentation associated with cortical microtubules has been observed in the anterior region of the spermatozoon as in most digenean species (Justine & Mattei, 1982; Miquel et al., 2006; Quilichini et al., 2007; Bâ et al., 2011; Ndiaye et al., 2012; Ndiaye et al., 2015a; Ndiaye et al., 2015b; Bakhoum et al., 2015a; Bakhoum et al., 2015b). However, the location of the external ornamentation distinguishes the spermatozoa of *O. dinema* and *P. selaris* from those of other digenean species. In fact, based on the diagram of the localization of the external ornamentation established by Quilichini et al. (2011a), the mature spermatozoon of *O. dinema* and *P. selaris* presents the type 1 of external ornamentation, i.e., located in proximal part of the anterior spermatozoon region. It is also interesting to note that in both *O. dinema* and *P. selaris*, the external ornamentation is extended from centriole level to the area containing the first mitochondrion. Such disposition of the external ornamentation is also reported in the monorchiid *M. parvus* but not in *H. atlanticus* (Table 1). This latter species seems to exhibit type 2 external ornamentation according to the diagram of Quilichini et al. (2011a).

#### Spine-like bodies

Since their first description by Miquel, Nourrisson & Marchand (2000) the spine-like bodies have frequently been reported in the mature spermatozoon especially in its anterior part. In the male gamete of *O. dinema* and *P. selaris*, spine-like bodies are present in the ornamented area associated with cortical microtubules. Moreover, in both monorchiid species the morphology of the spine-like bodies follows that reported in most digenean species i.e., appearance of a small vesicle interrupting the external ornamentation of the plasma membrane (Miquel et al., 2006; Quilichini et al., 2011b; Bakhoum et al., 2015b; Ndiaye et al., 2015a; Ndiaye et al., 2015b).

Within the Monorchiidae, the absence of spine-like bodies was mentioned in the male gamete of *M. parvus* and *H. atlanticus* (Levron, Ternengo & Marchand, 2004a; Diagne et al., 2015) (Table 1) hence the spine-like bodies are described here, for the first time, in the mature spermatozoon of monorchiid species.

Taking into account all these aspects, the presence or absence of spine-like bodies in the Monorchiidae needs more ultrastructural investigations.

#### Number of mitochondria

The presence of mitochondria in the mature spermatozoon is considered as a plesiomorphic character in the Digenea (Bakhoum et al., 2014) whereas in other groups, such as the Eucestoda, the absence of mitochondria has been highlighted as a synapomorphy (Justine, 1991a). Besides its interest in phylogenetic relationships, the other criterion related to mitochondria is their number.

Based on many cross- and longitudinal sections, two mitochondria are evident in the mature spermatozoon of *O. dinema* and *P. selaris*. The presence of two mitochondria have also been reported in *M. parvus*, *H. atlanticus* (Table 1) and in several species belonging, for instance, to the families Acanthocolpidae (Bakhoum et al., 2015a), Apocreadiidae (Kacem et al., 2010), Deropristidae (Foata, Quilichini & Marchand, 2007) or Notocotylidae (Ndiaye et al., 2003; Ndiaye et al., 2015b). Other digenean species possess one mitochondrion [e.g., *Carmyerius endopapillus* (Seck, Marchand & Bâ, 2008), *Wardula capitellata*; (Bakhoum et al., 2012b)] or three mitochondria [(e.g., *Anisocoelium capitellatum* (Ternengo et al., 2009), *Euryhelmis squamula* (Bakhoum et al., 2009).

In the present study, the position of the second mitochondrion is an ultrastructural characteristic that distinguishes the mature spermatozoa of *O. dinema* from those of *P. selaris*, *H. atlanticus* and *M. parvus*. The second mitochondrion appears after the disorganization of the first axoneme in *O. dinema*, whereas in the remaining monorchiid the appearance of the second mitochondrion is noted before the posterior extremity of the first axoneme.

#### Posterior spermatozoon characters

In Digenea the posterior spermatozoon extremity is morphologically variable and is, therefore, valuable in the establishment of spermatozoon models (Quilichini et al., 2010; Ndiaye et al., 2015b; Bakhoum et al., 2012a; Bakhoum et al., 2015a). In all monorchiid species described so far the posterior spermatozoon extremity contains a nucleus as in *O. dinema* and *P. selaris* (Table 1). This morphology of the posterior spermatozoon extremity is frequently reported in digenean (Kacem et al., 2010; Kacem et al., 2015a; Kacem et al., 2015b; Zhukova, Mordvinov & Kiseleva, 2014; Quilichini et al., 2015) and corresponds to the fasciolidean type or type 2 according to the diagram of Quilichini et al. (2010).

Other types of posterior spermatozoon extremities have been reported in digenean species. This is the case of mature spermatozoa exhibiting one axoneme as described in most microphalloid species (Bakhoum et al., 2012a; Miquel et al., 2013; Bruňanská et al., 2014) and opisthorchioid species (Quilichini et al., 2009; Foata et al., 2012; Zhukova, Mordvinov & Kiseleva, 2014). In the other hand, in the families Opecoelidae and Opistholebetidae (Miquel, Nourrisson & Marchand, 2000; Levron, Ternengo & Marchand, 2003; Levron, Ternengo & Marchand, 2004b; Quilichini et al., 2010; Quilichini et al., 2011b), there are mature spermatozoa showing only cortical microtubules in their posterior extremities.

### Phylogenetic perspectives of ultrastructural characteristics

The ultrastructural differences observed in the spermatozoa are phylogenetically informative in the Neodermata, particularly in the Cestoda and Monogenea (Justine, 1991b; Justine, 1998; Levron et al., 2010). In the Digenea a large amount of spermatological data accumulated over the recent years could serve as a complementary resource for understanding their phylogenetic relationships (Quilichini et al., 2010; Miquel et al., 2013; Bakhoum et al., 2015a; Diagne et al., 2015).

The phylogenetic relationships in the Monorchiidae and its systematic placement within the Digenea have been controversial. The Monorchiidae has been considered polyphyletic because of the great diversity exhibited in the morphology of its members (Madhavi, 2008). The most recent classification based on DNA sequences includes the Monorchiidae and Lissorchiidae in the superfamily Monorchioidea (Bray, 2008), which was included in the new suborder Monorchiata (Olson et al., 2003).

Here, the understanding of the relationships and phylogenetic affinities in Monorchiidae are attempted with ultrastructural characteristics of the spermatozoa, although there still remain some unexplored groups. The morphology of the spermatozoa described in *O. dinema*, *P. selaris* and other monorchiid species is similar in several points to that reported in the apocreadiid *Neoapocreadium chabaudi* (Kacem et al., 2010). The main similarities concern (1) the type of axoneme, (2) the morphology of both anterior and posterior spermatozoon extremities, (3) the presence of type 1 external ornamentation according to Quilichini et al. (2011a), (4) the two bundles of parallel cortical microtubules and (5) the presence of spine-like bodies.

Besides these similarities, it is interesting to note that the systematic position of the Apocreadiidae is unresolved. Some authors have grouped this family with the Haploporoidea and Monorchioidea (Cribb et al., 2001). Whereas other researchers suggested the removal of the Apocreadiidae from the Lepocreadioidea (see Bray & Cribb, 2012). Thus, to our knowledge no robust data are available to validate the phylogenetic position of the Apocreadiidae.

The present spermatological findings suggest, for the first time, a close relationship between the Monorchiidae and the Apocreadiidae based on ultrastructural characteristics of their mature spermatozoa. However, further studies are needed in order to support our hypothesis. Moreover, ultrastructural studies in the Lissorchiidae (probably a sister group) are also needed to test their close relationships with the Monorchiidae.

### Conclusion

The mature spermatozoa of *Opisthomonorchis dinema* and *Paramonorcheides selaris* share several ultrastructural features with those reported in *Monorchis parvus* and *Helicometroides atlanticus* (Table 1). These similarities allow us to define the type of spermatozoon in the Monorchiidae, which exhibits the following features:

– Presence of an anterior extremity showing two axonemes accompanied by cortical microtubules and external ornamentation,
– Presence of the association “external ornamentation+cortical microtubules”,
– Location of the external ornamentation corresponding to the type 1 according Quilichini et al. (2011a),
– Presence of two bundles of parallel cortical microtubules and two mitochondria,
– Posterior spermatozoon extremity containing only the nucleus according to Quilichini et al. (2010).

The model of mature spermatozoon described here for the monorchiid species is relatively similar to that reported to the apocreadiid *Neoapocreadium chabaudi* (Kacem et al., 2010). These similarities allow us to suggest a close relationship between the Monorchiidae and Apocreadiidae, although additional spermatological evidences are needed especially, from the unexplored taxa belonging to the families mentioned above and the Lissorchiidae.

The ultrastructural characteristics described in digenean and in particular, the Monorchiidae could be used as phylogenetic tools and when establishing spermatozoon models, given that they may allow the distinguishing of genera, families or superfamilies within the Digenea.

## Additional Information and Declarations

### Data Availability

The following information was supplied regarding data availability: The research in this article did not generate, collect or analyse any raw data or code.
